# Hydration with Mannitol and Dextrose May Promote Cisplatin-Induced Nephrotoxicity: Test of Five Protocols of Hydration during Cisplatin Therapy in Rat Models

**DOI:** 10.1155/2021/5547341

**Published:** 2021-10-04

**Authors:** Mohammad-Sedigh Khosravi, Alireza Samimiat, Bahar Mazaheri, Farzaneh Ashrafi, Ardeshir Talebi, Mehdi Nematbakhsh

**Affiliations:** ^1^Water & Electrolytes Research Center, Isfahan University of Medical Sciences, Isfahan, Iran; ^2^Department of Internal Medicine, Oncology-Hematology Section, Isfahan University of Medical Sciences, Isfahan, Iran; ^3^Department of Clinical Pathology, Isfahan University of Medical Sciences, Isfahan, Iran; ^4^Department of Physiology, Isfahan University of Medical Sciences, Isfahan, Iran; ^5^Isfahan^MN^ Institute of Basic & Applied Sciences Research, Isfahan, Iran

## Abstract

**Backgrounds:**

Cisplatin (CP) still is a novel choice for solid tumor therapy, but it is accompanied with the side effect of nephrotoxicity. Hydration may reduce the risk of CP-induced nephrotoxicity, while the issue is still challenging. In this study, five types of hydration protocols including saline, mannitol, dextrose saline, saline plus furosemide, and saline plus mannitol were examined in both sexes of rats during CP therapy.

**Methods:**

Seventy-six male and female Wistar rats in 14 groups of experiments were subjected to CP therapy, and five types of hydration protocols were implemented, and the induced nephrotoxicity was evaluated via biochemical markers, kidney function parameters, and pathology investigation.

**Results:**

Male and female rats had different responses to hydration protocol types. The higher mortality rate was seen in female rats that received mannitol or dextrose hydration types. In addition, the serum levels of blood urea nitrogen (BUN) and creatinine (Cr) and sodium excretion fraction (ENa%) increased and the clearance of Cr (ClCr) decreased significantly (*P* < 0.05) in female rats hydrated with saline plus furosemide or mannitol plus saline-treated groups. The worsened condition in male rats is observed in the mannitol hydration group with a significant decrease of ClCr and significant increase of serum BUN and Cr and ENa% (*P* < 0.05). The higher kidney tissue damage score (KTDS) in the mentioned groups verified the findings.

**Conclusion:**

Hydration with mannitol or dextrose promotes the risk of nephrotoxicity during CP therapy with more intensity on the female.

## 1. Introduction

Nowadays, cisplatin (CP) is recognized as a choice treatment for a wide range of solid tumors in clinic. In spite of its great effect on improvement of survival, it causes many complications such as nephrotoxicity. The renal manifestations of CP therapy may accompany with acute and chronic renal failure, tubular acidosis, or renal salt wasting [1]. Nephrotoxicity has still remained as the most common and important usage limiter of CP [2]. The kidney eliminates CP, while the main site of injury is in the renal epithelial cells of proximal tubule where the CP concentration increases to three times the serum concentration. Therefore, the nuclear and mitochondrial DNA injury and cells death (apoptosis) were performed, and furthermore, the reactive oxygen species generation and inflammatory mechanisms are also related in the CP-induced nephrotoxicity process [1, 3–5]. In addition, there are some risk factors for CP-induced nephrotoxicity including sex, history of renal dysfunction, drug dose, and the frequency of drug administration, older age, female gender, and hypoalbuminemia [1, 5, 6], while diabetes and organic cation transporter 2 (OCT2) polymorphisms are considered as decreased risks factors for CP-induced nephrotoxicity [1]. However, in the animal model, it was found that CP therapy promotes the induced nephrotoxicity in male more than female [6].

Several studies have been done in order to assess the protective effect of different synthetic and herbal antioxidants supplements against CP-induced nephrotoxicity [7–11]. In addition, other studies have been tried to alleviate the adverse effect of CP-induced nephropathy [12–14]. However, hydration and diuretics also devote to protect the kidney against CP-induced nephrotoxicity [15, 16]. By hydration with or without diuretic, the renal excretion is increased, and the contact duration of drug with the renal tubules is decreased, and the risk of CP-induced nephrotoxicity may attenuate, but the issue is still challenging. In this current study, five types of hydration protocols including saline, mannitol, dextrose saline, saline plus furosemide, and saline plus mannitol were examined in both sexes of rats during CP therapy, and the induced nephrotoxicity was evaluated.

## 2. Methods

### 2.1. Animals

Seventy-six female and male (209 ± 1.7 g) Wistar rats (Animal Center, Isfahan University of Medical Sciences, Isfahan, Iran) were used in this study. Animals were housed in the controlled conditions (12/12 light/dark cycles; 23–25°C). This study approved by Isfahan University of Medical Sciences Ethics Committee (IR.MUI.MED.REC.1399.707 for male rats and IR.MUI.MED.REC.1399.708 for female rats).

### 2.2. Experimental Design

The animals were divided randomly into 14 groups of experiments (groups 1–7 and groups 8–14 included male and female rats, respectively).

#### 2.2.1. Experimental Groups

(1)  Groups 1 (male, *n* = 6) and 8 (female, *n* = 6) are named NS + CP + NS. The animals in these groups initially received normal saline (NS, 15 ml/kg, ip), and 1 hour later, CP (7.5 mg/kg, ip) was administrated. Then, after 1 hour post-CP injection, the second injection of NS (15 ml/kg, ip) was done.
(2) Groups 2 (male, *n* = 6) and 9 (female, *n* = 6) are named M + CP + M, and groups 3 (male, *n* = 6) and 10 (female, *n* = 6) are named DS + CP + DS. The animals in these groups had the same regimen of group 1, but the M + CP + M groups received mannitol (M, 15 ml/kg, ip) and the DS + CP + DS received dextrose saline (DS, 15 ml/kg, ip) instead of NS.
(3) Groups 4 (male, *n* = 6) and 11 (female, *n* = 6) are named NS + CP + NS, F. The rats initially received NS (15 ml/kg, ip), and 1 hour later, CP (7.5 mg/kg, ip) was administrated. Then, after 1 hour post-CP injection, the combination of NS (12.5 ml kg, ip) and furosemide (F, 2.5 ml/kg, ip) was administrated.
(4) Groups 5 (male, *n* = 6) and 12 (female, *n* = 6) are named M, NS + CP + NS. The animals of these groups initially received combination of NS (7.5 ml/kg, ip) and M (7.5 ml/kg, ip), and 1 hour later, CP (7.5 mg/kg, .p) was injected. Then, after 1 hour post-CP injection, the NS (15 ml/kg, ip) was administrated.
(5) Groups 6 (male, *n* = 6) and 13 (female, *n* = 6) are named CP. The rats in these groups received CP (7.5 mg/kg, ip) alone without any hydration.
(6) Groups 7 (male, *n* = 4) and 14 (female, *n* = 4) are named NS. These rats had the regimen-like group 6 or 13, but they received NS (0.5 ml/kg, ip) instead of CP.

#### 2.2.2. Measurements

The animals were observed for period of 7 days, but before the end of experiment, they are kept in standard metabolic cages for 6 hours to collect urine. Finally, the blood samples were obtained by heart puncture under anesthesia, and the animals were sacrificed humanly. The kidneys were excised and weighted immediately to obtain the kidney weight (KW). The left kidney was used for histopathology investigations via hematoxylin and eosin (H&E) staining. Based on existence of vacuolization, dilatation, hyaline cast, debris, or degeneration, the renal damage was assigned as the kidney tissue damage score (KTDS), and it was scored from 1 to 4, while zero score was recorded for normal tissue [17, 18]. The right kidney was homogenized in specific volume of saline and centrifuged, and the supernatant was used for assessment of the kidney level of nitrite and malondialdehyde (MDA). The serum levels of creatinine (Cr), blood urea nitrogen (BUN), nitrite, MDA, and the Cr concentration in urine were measured. The serum level of nitrite was determined by the Griess method [19]. The urine and plasma sodium (Na) concentrations were also measured. The Cr clearance (ClCr), urine flow rate (UF), and the percentage of excretion Na fraction (ENa%) were also determined.

### 2.3. Statistical Analysis

Data are expressed as mean ± standard error of the mean. The levels of BUN, Cr, MDA, nitrite, UF, ClCr, ENa%, KW, and bodyweight (BW) change were compared between each two groups using the *t*-Student test. Comparison of the KTDS between the groups was assessed using the Mann–Whitney test. *P* < 0.05 was considered as significant.

## 3. Results

### 3.1. Animal Survival

The survival time (in days) for each group of male and female rats is given in Table 1. In general, male groups have more survival time rather than female groups. The groups of M + CP + M and DS + CP + DS that have the highest number of deaths in female sex showed a significant difference when compared with NS and CP groups. Furthermore, in male rats, there is the highest number of death in the M + CP + M group with survival time of 6.5 ± 0.2 days.

### 3.2. The Data for the Higher Mortality Rate Groups

According to survival time table (Table 1), the number of survived female rats in groups 9 (M + CP + M) and 10 (DS + CP + DS) which were hydrated with mannitol and dextrose, respectively, were less than 3 animals which are not enough to analyze. However, the data for BUN, Cr, KW, percentage change of bodyweight (% BW), and KTDS in one animal survived in group 9 (M + CP + M) were 553 mg/dl, 9.4 mg/dl, 1.10 g/100 g BW, −22.9%, and 4, respectively. The data for the mentioned parameter for the two animals survived in the group 10 (DS + CP + DS) were 628 ± 34.5 mg/dl, 7.6 ± 0.3 mg/dl, 1.10 ± 0.2 g/100 g BW, −15.7 ± 0.9%, and 4, respectively. These findings reveal the nonprotective role of M and DS hydration against CP-induced nephrotoxicity in female, while such observation was not seen in male.

### 3.3. The Effect of CP Alone on the Kidney

In order to determine the effect of CP alone, the data obtained in NS and CP groups (groups 6 and 7; male rats and groups 13 and 14; female rats) were compared statistically. The result is tabulated in Table 2, and the data revealed that CP increased significantly the serum levels of BUN, Cr, KW and KTDS which indicated kidney toxicity (Table 2).

### 3.4. The Effect of Hydration Protocols on CP-Induced Nephrotoxicity

**Figure 1:**
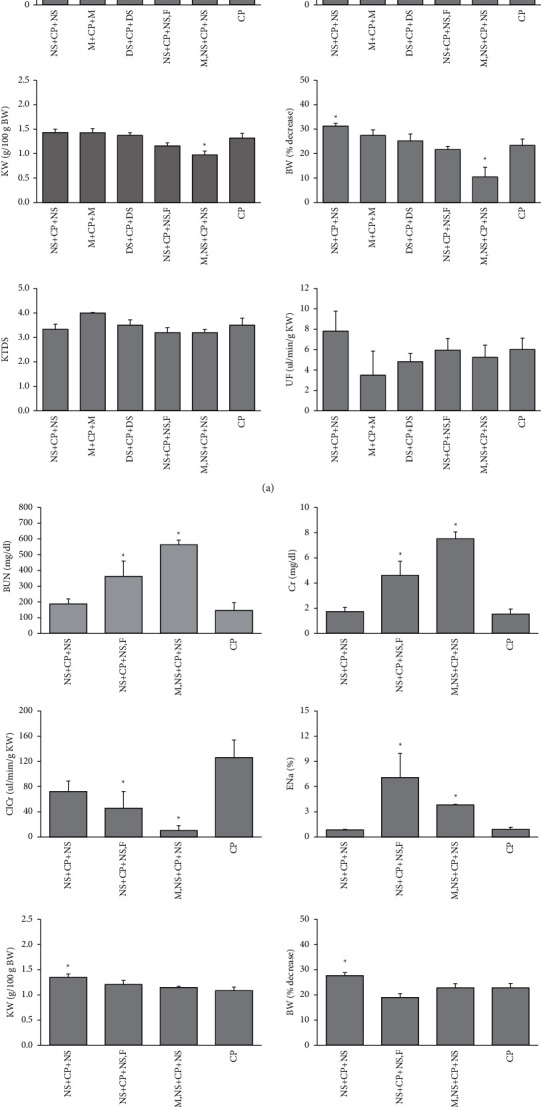
The serum levels of blood nitrogen urea (BUN) and creatinine (Cr), kidney weight (KW) per bodyweight (BW), percentage decrease of BW, kidney tissue damage score (KTDS), and urine flow rate (UF) among NS + CP + NS, M + CP + M (male), DS + CP + DS (male), NS + CP + NS, F, M, NS + CP + NS, and CP groups in male (a) and female (b) rats. ^∗^Significant difference from the CP group (*P* < 0.05) by the *t*-Student test.

In male rats, the serum levels of BUN and Cr and ENa% were increased significantly (*P* < 0.05) in the M + CP + M group, while these increased insignificantly in the DS + CP + DS group when compared with the CP-alone treated group. In addition, the ClCr also was decreased statistically in the M + CP + M group, as shown in Figure 1(a). The normalized KW and percentage change of BW in the M, NS + CP + CP group have the lowest value compared with the CP alone treated group (*P* < 0.05). Also, a lower UF and a higher KTDS were detected insignificantly in M + CP + M groups, as shown in Figure 1(a).

In female rats, the serum levels of BUN and Cr and ENa% were increased significantly (*P* < 0.05) in M, NS + CP + NS and NS + CP + NS, F groups when compared with the CP alone treated group, as shown in Figure 1(b). In addition, ClCr also decreased significantly in NS + CP + NS, F and M, NS + CP + NS groups from the CP group (*P* < 0.05). The increase of KW and more BW lost were observed in the NS + CP + NS group from the CP group (*P* < 0.05), and a lower UF was collected significantly in NS + CP + NS and NS + CP + NS,F groups compared to the CP alone treated group (*P* < 0.05).

**Figure 2:**
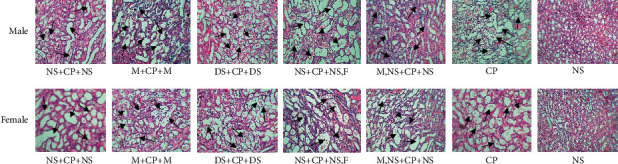
The samples images (×100) of kidney tissues in all experimental groups. The arrows indicate the tissue damage including vacuolization, dilatation, hyaline cast, debris, or degeneration.

Finally, the serum and kidney levels of nitrite and MDA in both male and female rats treated with different hydration protocols are given in Table 3. The sample image of kidney tissue for each experimental group is shown in Figure 2.

## 4. Discussion

CP is used for a wide range of cancers during the chemotherapy process, but due of its side effect of nephrotoxicity, its use is limited. Proper hydration is proposed to reduce the CP-induced nephrotoxicity. In this study, the effectiveness of five types of hydration methods was investigated. The findings indicated that hydration with mannitol or dextrose promote the mortality rate in female rats. The rats that received mannitol have the highest mortality rate. Only 1 female rat (16%) and 3 male rats (50%) survived at the end of the experiment. In addition, the mortality rate in the female dextrose-treated group (group 10) was 66%.

CP filters via the glomerulus, and it excretes through the renal tubules [5]. ClCr is the best index to estimate the glomerular filtration rate (GFR), and the serum level of Cr has been increased when GFR has been attenuated [16, 20]. These two parameters and others indexes such as serum levels of BUN and Cr, KW and KTDS altered in CP alone treated groups of male and female rats which verified the effect of CP. CP alone also decreased the kidney level of nitrite and increased the serum level of nitrite as other studies [21–23].

Reviews of past studies illustrate controversial results about mannitol. Some of them reported that mannitol has a protective effect on the kidneys by its diuretic mechanism and reducing CP concentration [24, 25]. On the contrary, some others studies declared no protective effect for mannitol, or even in confirmation with our study, they found mannitol to be significantly more nephrotoxic [16, 26, 27]. Morgan et al. indicated that forced diuresis increased the risk of acute kidney injury due to volume decreasing [27]. Shi et al. demonstrated that high dose of mannitol caused stress oxidative, destroyed cellular cytoskeleton, and promoted apoptosis [28]. Also, it has been indicated that mannitol causes osmotic nephrosis and lead to acute renal failure [29, 30]. Some studies also have shown the benefits of mannitol on lowering the CP-induced nephrotoxicity [16, 27]. However, the subsequent study has not reported a significant difference between hydration plus or without mannitol [31, 32]. There also is concern about the use of mannitol due to vasoconstriction feature and the possibility of prerenal azotemia [33, 34].

The nephroprotective effect of mannitol and furosemide have also been compared. Santoso et al. concluded that furosemide-saline has more nephroprotective effect compared with mannitol [16], while other studies considered no significant difference in their protective role [26]. However, in our study, no significant protective effect was observed for furosemide-saline hydration in male rats, but the serum levels of BUN and Cr increased significantly when compared with the CP group in female rats. The protective effect of furosemide like mannitol is still debatable [16], while the neutral effect and adverse effect of furosemide on the kidney also are reported [35–37].

An increase in the glucose level to perform a suitable diuresis is another issue for challenging. Diabetes has been considered as one of the risk factors for various cancers such as colon, chest, pancreas, and liver, but it plays a protective role in reducing the toxic effects of CP. Intensity and duration of diabetes mellitus have a direct relationship with the severity of kidney toxicity, and the protective effects are also reduced by controlling diabetes [38]. Therefore, the protective effects of dextrose saline (normal saline with glucose) with the likelihood of a similar role of diabetes also need to be considered.

Finally, in the current study, we reported the data for male and female rats separately, and some differences were seen without any analysis. However, it is well documented that CP-induced nephrotoxicity is gender related [6]. Pezeshki et al. mentioned that administration of estrogen increases severity of CP-induced nephrotoxicity [39], so the increase in the serum level of BUN and Cr and other factors in some female groups may be due to estrogen hormone. Generally, it seems that gender is an important factor affecting the hydration process during CP therapy.

## 5. Conclusion

Although mannitol and dextrose in clinical protocols for the reduction of CP-induced nephrotoxicity may be suggested, however, this study not only shows that these two hydration protocols do not protect the kidney but also they are harmful and the use of these substances need to be reconsidered seriously in male and female genders. It seems that hydration with saline alone is more safe than other protocols investigated in this study.

## Figures and Tables

**Table 1:** The mean values of survival time for each group of male and female rats during the experiment.

Group	*n*	Group name	Number of death animals (male)						Survived	Survival time (day)	Group	*n*	Number of death animals (female)						Survived	Survival time (day)	*P*_Wilcoxon_
		Day	1	2	3	4	5	6					1	2	3	4	5	6			
1	6	NS + CP + NS	—	—	—	—	—	—	6	7.0 ± 0.0	8	6	—	—	—	—	—	—	6	7.0 ± 0.0	1.0
2	6	M + CP + M	—	—	—	—	—	3	3	6.5 ± 0.2^a,b^	9	6	—	—	—	—	—	5	1	6.2 ± 0.2^a,b^	0.4
3	6	DS + CP + DS	—	—	—	—	—	—	6	7.0 ± 0.0	10	6	—	—	—	—	—	4	2	6.3 ± 0.2^a,b^	0.06
4	6	NS + CP + NS,F	—	—	—	—	—	1	5	6.8 ± 0.2	11	6	—	—	—	—	—	—	6	7.0 ± 0.0	0.7
5	6	M, NS + CP + NS	—	—	—	—	—	—	6	7.0 ± 0.0	12	6	—	—	—	—	—	1	5	6.8 ± 0.2	0.7
6	6	CP	—	—	—	—	—	—	6	7.0 ± 0.0	13	6	—	—	—	—	—	1	5	6.8 ± 0.2	0.7
7	4	NS	—	—	—	—	—	—	4	7.0 ± 0.0	14	4	—	—	—	—	—	—	4	7.0 ± 0.0	1.0
*P*_Kruskal–Wallis H_										0.033	*P*_Kruskal–Wallis H_									0.002	

The small alphabetic letters indicate a significant difference from (a) NS and (b) CP (*P* < 0.05). Each two groups of male and female that received a similar hydration protocol was compared using Mann–Whitney U analysis. Refer the text for group treatment. The comparison of survival time between each sex was performed using the Wilcoxon test.

**Table 2:** The effect of cisplatin (CP).

Parameter/group	Male			Female		
	NS	CP	*P*	NS	CP	*P*
BUN (mg/dl)	24.0 ± 0.4	128.4 ± 42.6	0.06	21.2 ± 1.2	146.0 ± 49.2	0.04
Cr (mg/dl)	0.4 ± 0.0	1.4 ± 0.4	0.09	0.57 ± 0.0	1.5 ± 0.4	0.05
KW (g/100 g BW)	0.7 ± 0.1	1.3 ± 0.0	0.01	0.6 ± 0.0	1.0 ± 0.0	<0.0001
BW(% change)	3.8 ± 2.0	−23.3 ± 2.5	<0.0001	11.5 ± 1.1	−22.8 ± 1.7	<0.0001
KTDS	0.5 ± 0.2	3.5 ± 0.2	0.02	0.2 ± 0.2	3.4 ± 0.6	0.01
UF (*μ*l/min g kW)	5.5 ± 2.1	6.0 ± 1.1	0.85	6.9 ± 2.0	8.8 ± 1.8	0.52
ClCr (*μ*l/min g kW)	530.1 ± 153.0	109.5 ± 34.0	0.03	334.5 ± 69.2	125.9 ± 28.0	0.03
ENa (%)	1.0 ± 0.2	2.6 ± 1.3	0.29	0.91 ± 0.24	1.0 ± 0.3	0.78
SNitrite (*μ*mol/L)	10.52 ± 1.2	13.37 ± 3.6	0.52	10.28 ± 1.4	24.58 ± 3.7	0.01
SMDA(*μ*mol/L)	4.9 ± 0.1	3.2 ± 0.2	<0.0001	3.8 ± 0.24	4.1 ± 0.15	0.35
KNitrite (*μ*mol/g tissue)	182.6 ± 4.2	148.0 ± 9.0	0.01	187.6 ± 19.7	103 ± 7.0	<0.0001
KMDA (nmol/g tissue)	64.2 ± 2.7	29.1 ± 1.2	<0.0001	65.9 ± 4.1	58.1 ± 11.8	0.55

*P* values are obtained by the *t*-Student test. BUN, blood urea nitrogen; Cr, creatinine; KW, kidney weight; BW, bodyweight; KTDS, kidney tissue damage score; UF, urine flow rate; ClCr, clearance of Cr, ENa%, excretion fraction of sodium; SMDA and KMDA, serum and kidney levels of malondialdehyde; serum and kidney levels of nitrite in normal saline (NS) and CP-treated groups of male and female rats.

**Table 3:** Serum and kidney levels of nitrite and MDA in both male and female rats treated with different hydration protocols.

Group/parameter	Male				Female			
	Snitrite (*μ*mol/l)	SMDA (*μ*mol/l)	Knitrite (*μ*mol/g tissue)	KMDA (nmol/g tissue	Snitrite (*μ*mol/l)	SMDA (*μ*mol/l)	Knitrite (*μ*mol/g tissue)	KMDA (nmol/g tissue
NS + CP + NS	10.4 ± 1.1	3.1 ± 0.2	89.5 ± 11.9^*∗*^	21.7 ± 4.5	36.7 ± 5.4	1.1 ± 0.2^*∗*^	106.0 ± 6.3	26.7 ± 3.0^*∗*^
M + CP + M	16.3 ± 5.3	2.2 ± 0.2^*∗*^	90.8± ± 15.7^*∗*^	28.2 ± 6.2	—	—	—	—
DS + CP + DS	22.1 ± 6.5	2.8 ± 0.6	122.2 ± 8.1^*∗*^	24.4 ± 3.8	—	—	—	—
NS + CP + NS, F	10.2 ± 1.6	2.6 ± 0.4	123.3 ± 9.5^*∗*^	38.3 ± 2.2^*∗*^	37.5 ± 12.5	2.5 ± 0.4^*∗*^	130.2 ± 14.8	41.1 ± 9.9
M, NS + CP + NS	14.5 ± 3.5	3.3 ± 0.1	152.1 ± 4.8	49.0 ± 6.3^*∗*^	36.4 ± 21.2	4.7 ± 0.9	109.9 ± 7.9	49.5 ± 19.4
CP	13.4 ± 3.6	3.3 ± 0.2	148.0 ± 9.1	29.2 ± 1.2	24.6 ± 3.8	4.1 ± 0.2	103.7 ± 7.0	58.1 ± 11.8

^*∗*^Significant difference from CP (*P* < 0.05) obtained by the *t*-Student test.

## Data Availability

The data used to support the findings of this study are available from the corresponding author upon request.
